# Low Molecular Weight Heparin-Induced Skin Necrosis: A Case Report

**DOI:** 10.1155/2011/857391

**Published:** 2011-05-30

**Authors:** Anastasios Katsourakis, George Noussios, George Kapoutsis, Efthimios Chatzitheoklitos

**Affiliations:** ^1^Department of Surgery, “Agios Dimitrios” General Hospital of Thessaloniki, 54627 Thessaloniki, Greece; ^2^Department of Physical Education (Serres), “Aristotelian” University of Thessaloniki, 54006 Thessaloniki, Greece

## Abstract

Low molecular weight heparins (LMWHs) are the standard agents used for antithrombotic therapy and prophylaxis. Despite their widespread use, reports about adverse effects from LMWHs are very scarce. Heparin-induced skin necrosis at the injection site is a rare adverse effect, more commonly associated with unfractionated heparin (UFH) rather than with LMWH, while its mechanism remains unclear. This paper deals with the enoxaparin induced skin necrosis.

## 1. Introduction

Heparin preparations are important anticoagulants and have been successfully used for the prevention and treatment of thromboembolic disorders for many years in most surgical units. We report the rare occurrence of skin necrosis that developed after enoxaparin administration. After a Medline literature search, we have found 25 cases of skin necrosis associated with LMWH until May 2009.

## 2. Case Report

On September 2007, a 76-year-old male patient was admitted to our hospital with pain located at the right hypochondrium and epigastrium. Routine laboratory testing showed an increased white blood cell account: 30.900 with 87.8% neutrophils and high levels of amylase 2076. He underwent ECHO of the abdomen which revealed cholelithiasis and pancreatitis.

The drug history of the patient revealed the intake of Lipitor (10 mg Atorvastatin) and Co-Renitec (20 mg Enalapril + 12.5 mg Hydroclorothiazide). Thrombophylaxis was started with LMWH (Enoxaparin Sodium, 40 IU s.c./24 h). The injection sites were located beside the umbilicus, in the abdominal wall.

**Figure 1:**
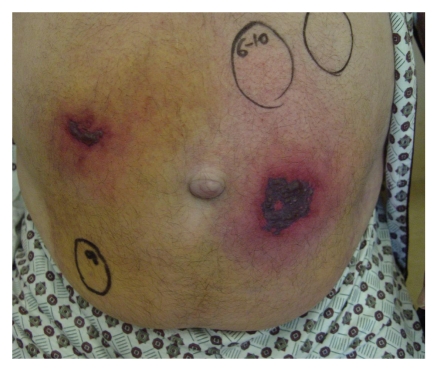
Macroscopic appearance of the lesion.

After 10 days of LMWH administration, we observed 2 progressive, painful erythematous lesions at the injection sites, and the next day, central necrosis of the lesions developed, with a clear line of demarcation between the affected area and surrounding tissue (the diameter of the lesion was 20 cm approximately 5 cm and 3.5 cm in diameter) (Figure 1).

The histopathological examination revealed necrosis of the surface skin as well as clots in small blood vessels with inflammatory changes of the deeper skin.

Coagulation tests and platelet count were normal. Heparin platelet factor-4 (HPF-4) antibody testing was negative by enzyme-linked immunosorbent assay (ELISA). The patient did not have thrombocytopenia or thrombotic complications. He had also normal plasma concentrations of protein C and protein S. No further platelet aggregation studies carried out in this patient. The development of this localized side effect led to the discontinuation of treatment with enoxaparin. We decided to switch from anticoagulant therapy to Nadroparin until the patient discharges. Tissue was taken for culture as well as muscle-skin biopsy, but both proved to be negative. The following course was uneventful, and the necrotic plaques disappeared within a week.

## 3. Discussion

Heparin-induced skin necrosis is an infrequently localized side effect that typically affects middle-aged women with a history of thrombotic disease [1]. The skin necrosis occurs between day 5 and 11 following initiation of therapy [2]. It may also occur immediately if the patient has been previously sensitized or may rarely be delayed for several months after treatment onset. 

The first symptoms are usually described as erythematous, subcutaneous lesions with oedema, and pain at the injection sites. Subsequently, bullous transformation is observed before full-skin necrosis occurred [3]. The size of the necrotic areas varied, but generally, the lesions appeared to be small and circumscribed, with a maximum diameter of a few centimeters. The lesion occurred locally at the injection site (abdominal wall, thigh, and arm). Less frequently, other anatomical areas far from the injection site were also affected. These distant manifestations were apparently random in distribution.The lesions are manifested by the sudden development of painful, erythematous, sharply demarcating plaques progressing to purpuric plaques, which tend to be necrotic within a short time.

The proposed mechanisms inducing heparin-induced skin necrosis are considered: immunologically mediated either via intravascular thrombosis resulting from heparin induced immune aggregation of platelets (Heparin-induced thrombocytopenia syndrome, HIT) or an Arthus-type reaction due to formation of antigen-antibody complexes in cutaneous blood vessels (Type III hypersensitivity syndrome) or an incorrect technique of injection. Lastly, fat tissue may have poor blood circulation, and this may result in the heparin persisting at the injection site and causing further damage [4].

Discontinuance of the heparin injections promptly leads to recovery. Wound care involves cleaning and dressing the areas of skin loss, with appropriate pain relief. Sometimes surgery is required to remove the dead skin and a skin graft may be performed if this is extensive, resulting in longer prolonged recovery time. If anticoagulation is still required, then an alternative drug should be used, and this may include aspirin, warfarin, hirudins, or unfractionated heparin, depending on the cause of the heparin necrosis. If HIT is excluded a change in heparin type may be used safely [5].

Heparin necrosis may rarely be fatal due to complications of large areas of skin loss in severe cases or, if heparin is not discontinued immediately and replaced by an appropriate anticoagulant in HIT, due to clots developing internally. 

The diagnosis is usually suspected clinically, but a skin biopsy may be performed. Histopathology shows death of the surface skin and sometimes clots or inflammation in small blood vessels of the deeper skin [6], as it was in our case.

## 4. Conclusion

Heparin-induced skin necrosis occurring after administration of a LMWH is rare, and several path mechanisms could be involved. However, early recognition of the condition is vital as continued treatment with heparin may precipitate life-threatening complications in other organ systems.
